# Early school failure predicts teenage pregnancy and marriage: A large population-based cohort study in northern Malawi

**DOI:** 10.1371/journal.pone.0196041

**Published:** 2018-05-14

**Authors:** Judith R. Glynn, Bindu S. Sunny, Bianca DeStavola, Albert Dube, Menard Chihana, Alison J. Price, Amelia C. Crampin

**Affiliations:** 1 Faculty of Epidemiology and Population Health, London School of Hygiene & Tropical Medicine, London, United Kingdom; 2 Malawi Epidemiology and Intervention Research Unit, Chilumba, Malawi; Tulane University School of Public Health and Tropical Medicine, UNITED STATES

## Abstract

**Background:**

School dropout has been linked to early pregnancy and marriage but less is known about the effect of school performance. We aimed to assess whether school performance influenced age at sexual debut, pregnancy and marriage, and from what age school drop-out and performance were associated with these later life events.

**Methods:**

Data from 2007–2016 from a demographic surveillance site in northern Malawi with annual updating of schooling status and grades, and linked sexual behaviour surveys, were analysed to assess the associations of age-specific school performance (measured as age-for-grade) and status (in or out of school) on subsequent age at sexual debut, pregnancy and marriage. Landmark analysis with Cox regression was used to estimate hazard ratios of sexual debut, pregnancy and marriage by schooling at selected (landmark) ages, controlling for socio-economic factors.

**Results:**

Information on at least one outcome was available for >16,000 children seen at ages 10–18. Sexual debut was available on a subset aged ≥15 by 2011. For girls, being out of school was strongly associated with earlier sexual debut, pregnancy and marriage. For example, using schooling status at age 14, compared to girls in primary, those who had dropped out had adjusted hazard ratios of subsequent sexual debut, pregnancy and marriage of 5.39 (95% CI 3.27–8.86), 2.39 (1.82–3.12), and 2.76 (2.08–3.67) respectively. For boys, the equivalent association with sexual debut was weak, 1.92 (0.81–4.55), but that with marriage was strong, 3.74 (2.28–6.11), although boys married later. Being overage-for-grade was not associated with sexual debut for girls or boys. For girls, being overage-for-grade from age 10 was associated with earlier pregnancy and marriage (e.g. adjusted hazard ratio 2.84 (1.32–6.17) for pregnancy and 3.19 (1.47–6.94) for marriage, for those ≥3 years overage compared to those on track at age 10). For boys, overage-for-grade was associated with earlier marriage from age 12, with stronger associations at older ages (e.g. adjusted hazard ratio 2.41 (1.56–3.70) for those ≥3 years overage compared to those on track at age 14). For girls ≥3 years overage at age 14, 39% were pregnant before they were 18, compared to 18% of those who were on track. The main limitation was the use of reported ages of sexual debut, pregnancy and marriage.

**Conclusions:**

School progression at ages as young as 10 can predict teenage pregnancy and marriage, even after adjusting for socio-economic factors. Early education interventions may reduce teenage pregnancy and marriage as well as improving learning.

## Background

Improving education is one of the Sustainable Development Goals, and underlies others: increasing education improves health, reduces poverty and helps gender equality [1]. For girls there are also major benefits for the next generation: half of the reduction in under-5 mortality achieved in the last 30 years may be attributable to increased maternal education [2]. There are also strong links to sexual health: education level is associated with age at first sex, condom use and HIV risk [3, 4].

Initial primary school enrolment is high in most countries, and often similar for boys and girls, but increasing dropout of girls in adolescence is a major and wide-spread problem [5]. Since schooling often starts late and grades are repeated, dropout in adolescence frequently means dropout before the end of primary school, as well as the loss of opportunities for secondary schooling and tertiary education.

The relationship between sexual behaviour and school dropout is complex. Most data on the association between schooling and sexual behaviour come from cross-sectional studies, making it difficult to distinguish cause and effect [4, 6]. Being out of school can lead to risky sexual behaviour, pregnancy and marriage, but unintended pregnancies and early marriage can lead to school dropout [5, 7]. Compared to out-of-school adolescents, those in school are less likely to have sex, have multiple life partners or have frequent sex [4]. Adolescents in school and performing better at school may have a higher perception of risk associated with early sexual debut, and higher aspirations for their future than their non-school going peers [6, 7]. For those in school, sexual activity poses a high opportunity cost, with unintended pregnancies and marriage as a deterrent to achieving educational goals. Those out of school may consider sexual activity desirable, potentially bringing marriage and financial security for the future.

Both school dropout and early sex, pregnancy and marriage are influenced by the same underlying factors, including poverty, poor school performance, absenteeism, school characteristics, and peer, family and community pressures and expectations [8–13]. High costs of schooling, lack of school infrastructure (from toilets to textbooks), and poor school performance may precipitate disinterest in school, which promotes risky sexual behaviour [14], and early school exit. Randomised trials in Kenya [15] and southern Malawi [16] suggest uniform provision and cash transfer can reduce school dropout, pregnancy and sexually transmitted infection rates, strengthening evidence that poverty underlies both outcomes, and that being in school is “protective” [7].

A review of determinants of adolescent sexual health in developing countries showed that school performance (high grade-point averages) and high levels of motivation to continue schooling provided protective effects for adolescents [17]. In South Africa, falling behind in school was the strongest risk factor for giving birth within the following two years [18]. The few longitudinal studies generally involve teenagers [11, 15, 16, 18], and it is unclear from what age school failure predicts subsequent life trajectories.

In Malawi school dropout is high and learning outcomes poor: the 2010 World Bank report on the education system estimated that only 52% of children completed 6 years of primary school compared to an average of 61% for sub-Saharan Africa, and test scores for English and Maths were among the lowest in the region [19]. A quarter of young adults do not have even basic literacy skills [20]. Malawi also has high rates of child marriage (42% of girls married by age 18): the constitution was amended to raise the age of marriage from 15 (with parental consent) to 18 in February 2017 [21].

In Karonga district, northern Malawi, the site of the current study, the proportions completing primary are better than the national average but still poor [22]. We have previously shown that girls drop out of school earlier than boys, and half of girls (and 8% of boys) reported pregnancy or marriage as the main reason for leaving school [22]. We have also shown that falling behind in school, measured by being increasingly overage for the school grade, is common, and is strongly associated with dropout [23]. In this paper we examine the associations between falling behind in school (age-for-grade) and school dropout with subsequent sexual debut, teenage pregnancy and marriage. We use a landmark approach (detailed below) and show that school performance at ages as young as 10 years predicts age at pregnancy and marriage.

## Methods

The Karonga Prevention Study Demographic Surveillance Site in northern Malawi covers a rural population of 35,000 people, collecting data, since 2002, on births and deaths monthly, with annual censuses to update migrations [24]. Linked surveys collect detailed household and individual socio-economic, schooling, demographic and behavioural data. Schooling data, including grade attainment, have been collected annually since 2007. Household-level socioeconomic data were collected annually between 2007–2011, and 2013–2016. Sexual behaviour data including age at first sex were collected on those aged 15 and over in three survey rounds between 2008 and 2011 [25]. Age at first pregnancy and marriage was collected in the sexual behaviour surveys and, from October 2013, with the demographic data for those aged 12 and over [24].

Ethics approval for the demographic surveillance and sexual behaviour studies was obtained from the National Health Sciences Research Committee in Malawi (#419) and Research Ethics Committee of the London School of Hygiene and Tropical Medicine. For the demographic surveillance, before the start of the study the Traditional Authority, village headmen and traditional advisors for the study area were informed about the study aims and the nature of the data to be collected, and their approval and verbal consent sought. All household members were given a similar explanation and interviews were only conducted if verbal consent was given by the household head and by the respective household members. The consent for the demographic surveillance was recorded by the interview sheet being filled. Refusals were recorded in field registers. During the baseline census 15 households did not provide verbal consent and were therefore excluded. The sociodemographic data for this study come from the basic demographic surveillance for which the ethics committees agreed that written consent was not needed. For the sexual behaviour surveys individual written informed consent was sought.

In this analysis we assessed the association of schooling performance and status at different ages on the subsequent risk of sexual debut, pregnancy and marriage. Exposures were defined as: current age-for-grade (the number of years a child is overage for their grade, among those in school), and current schooling status (in primary, in secondary, dropped out during primary, dropped out after primary). In Malawi primary school has eight grades and secondary school four forms. Schooling starts, theoretically, at age 6, so a child progressing optimally would spend one year at each level and finish primary at age 14 and secondary at 18. Children with poor performance are required to repeat the year. Some children start late, and many repeat levels, so they become increasingly over-age for their grade [23]. In this population few start late: about 92% start at 6 or younger, and only 1% start older than 7. Primary school has no fees. Secondary school has fees, and places are restricted so there is a bottle-neck at the end of primary [26] and children may repeat the final year to improve their results. As academic failure and under-achievement are major causes for repetition, age-for-grade is a marker of school progress.

We used a landmark approach [27] because both exposures and risks change quickly with age and we aimed to examine the effect of earlier schooling on life transitions (sexual debut, pregnancy and marriage). With this method, using yearly landmarks, the situation for each participant is taken at each single year of age and the subsequent rate of the outcomes examined. Individuals in the demographic surveillance could age into and out of the cohort, and were included in the analysis for each year in which they were aged 10–18 since 2007, with follow-up to age 20 or 25 (see below). For each landmark analysis, the rates measured are conditional on the exposure (e.g. age-for-grade) and confounders (e.g. living arrangements) at the landmark age, ignoring any change of status thereafter. Because age at sexual debut, pregnancy and marriage were reported by year, a random fraction of a year was added to the ages to convert them to dates.

Survival analysis with Cox regression models was used to estimate hazard ratios for each of the outcomes (sexual debut, pregnancy and marriage). For each landmark analysis, those who had already experienced the event by the landmark age were excluded, and individuals were included from the date at which they were first seen at that landmark age. Individuals were kept in the analysis until they experienced the event of interest, or the last date at which they were asked about the outcome (the date of the last interview at which the relevant data were recorded), or they reached age 20 or 25. For girls all analyses were censored at age 20 as the interest was in early pregnancy and marriage. For boys marriage is rare under 20 years so the time period was extended to age 25.

Analyses were done with and without adjusting for confounders. For clarity the same set of confounders were included in all analyses. These were: education of parents, vital status of parents, living arrangements (household size, number of children aged 0–5 years in household, living with parents), sex of head of household, socioeconomic status (as five levels from principal component analysis of household assets), year of interview. The proportion with missing values for these confounders was very low (<1%) for all except asset score (~7%). Complete case analysis was used for the Cox regression analyses, thereby excluding those with missing data. Other possible confounders were examined: dwelling score (which was only available until 2011), age of parents at birth, and first born or subsequent child. Further adjustment for these variables did not affect results and because they would have added to the proportion with missing values they are not included. To assess whether associations with age-for-grade were explained by the age at starting school, we re-ran the analyses adding this variable as a possible confounder.

There was some evidence of departure from proportionality for analyses with age-for-grade (girls age 12–14 and boys at age 13 only), and a larger departure from proportionality for analyses with schooling status at all ages, with the hazard ratios of the outcomes decreasing with age due to the high initial hazard of the outcomes after school dropout. For simplicity of comparison across landmark analyses, we report the estimated hazard ratios obtained under the proportional hazards assumption, noting that these estimates are averages of time-varying hazard ratios over the follow-up time.

## Results

In this open cohort, information on at least one outcome (age at sexual debut, first pregnancy or first marriage) was available for more than 16,000 children with schooling information at ages 10–18 years. Few children were two or more years over-age for their grade when younger than 10 years, and very few children dropped out of school before age 13, so the analyses of school progression and schooling status were restricted to those aged ≥10 and ≥13, respectively.

Information on age at first marriage was available for 8576 girls and 7751 boys, on pregnancy for 6999 girls, and on sexual debut (which was only asked for those aged ≥15 between 2008 and 2011) for 2361 girls and 2207 boys. The numbers available for each landmark age analysis are different: those who had already had the outcome are excluded; there are almost no data on sexual debut for those with schooling data at age <12 years; and data on pregnancy and marriage are missing for some individuals, due to age eligibility, timing of the surveys or lack of time for follow-up surveys for those seen in the last year.

For example, for girls, there were 4592 seen at age 10, 3811 at age 14, and 3258 at age 18. At age 14: 890 (23%) girls had data on sexual debut and 56 had already had sex. After excluding those with missing data on confounders, 817 were included in the school status analysis, and 777 in the age-for-grade analysis (which excluded those who had already left school). Similarly, for girls at age 14, 2703 (71%) had data on first pregnancy, 40, had already been pregnant, 2508 were included in the schooling status analysis and 2408 in the age-for-grade analysis; and 2978 (78%) had data on marriage, 67 had already been married, 2744 were included in the school status analysis and 2644 in the age-for-grade analysis.

The rates of sexual debut, first pregnancy and first marriage by schooling status, age-for-grade and the potential confounders are shown in S1 Table for landmark age 14 for girls. At this age very few children had reached secondary school, and few had already experienced any of the outcomes (as described above). As well as associations with schooling status and age-for-grade, discussed below, sexual debut, pregnancy and marriage tended to be later (shown as lower rates) in those with higher socio-economic status, living with their parents, and with more educated parents (for pregnancy and marriage only). Although some children started school young, because of early repetitions few children were underage for their grade (5% by age 10, 2% by age 14), so they are grouped with those at the correct age-for-grade for the analyses.

**Fig 1:**
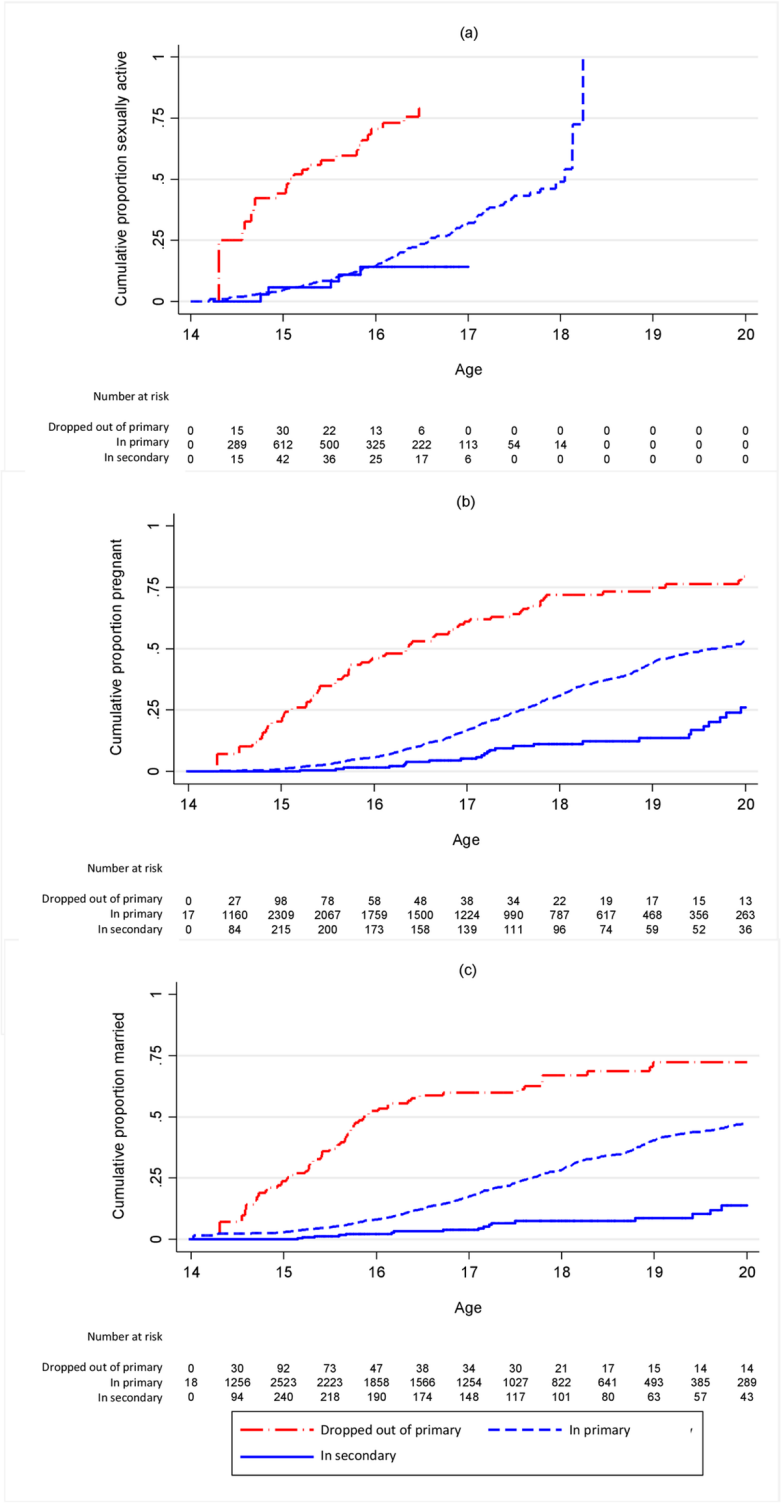
Cumulative proportion ever (a) sexually active (b) pregnant or (c) married by schooling status of girls at landmark age 14. Restricted to those who had not yet had the outcome in question.

**Fig 2:**
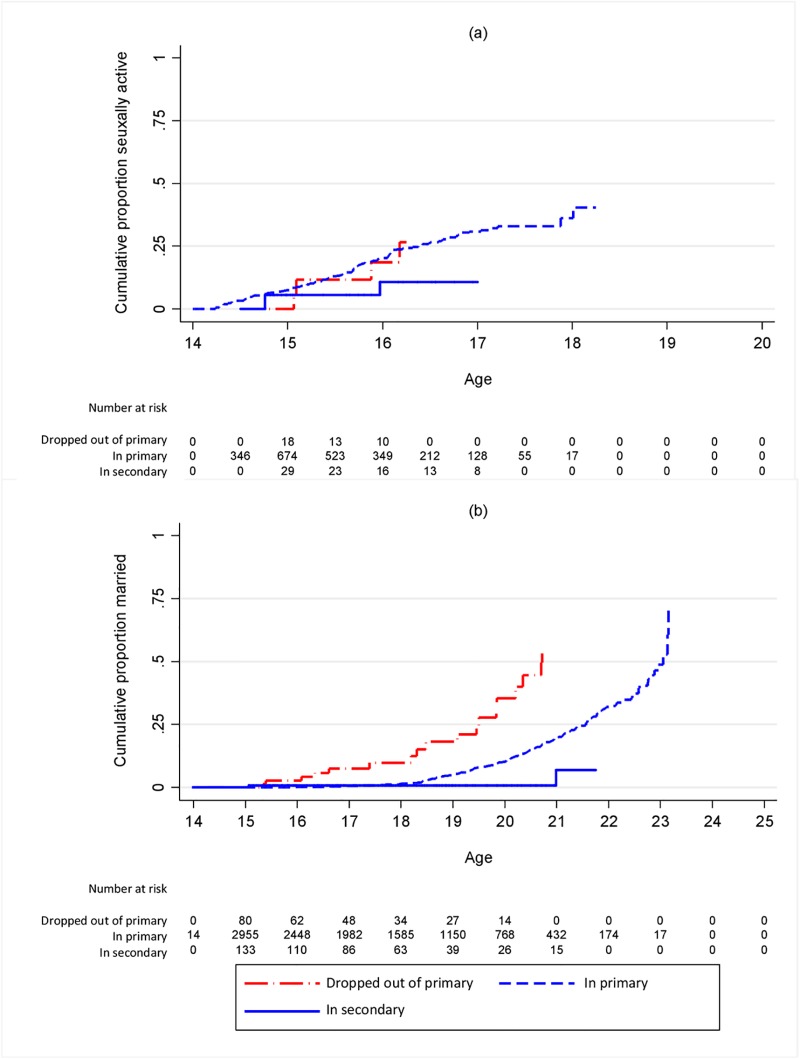
Cumulative proportion ever (a) sexually active (b) married by schooling status of boys at landmark age 14. Restricted to those who had not yet had the outcome in question.

**Fig 3:**
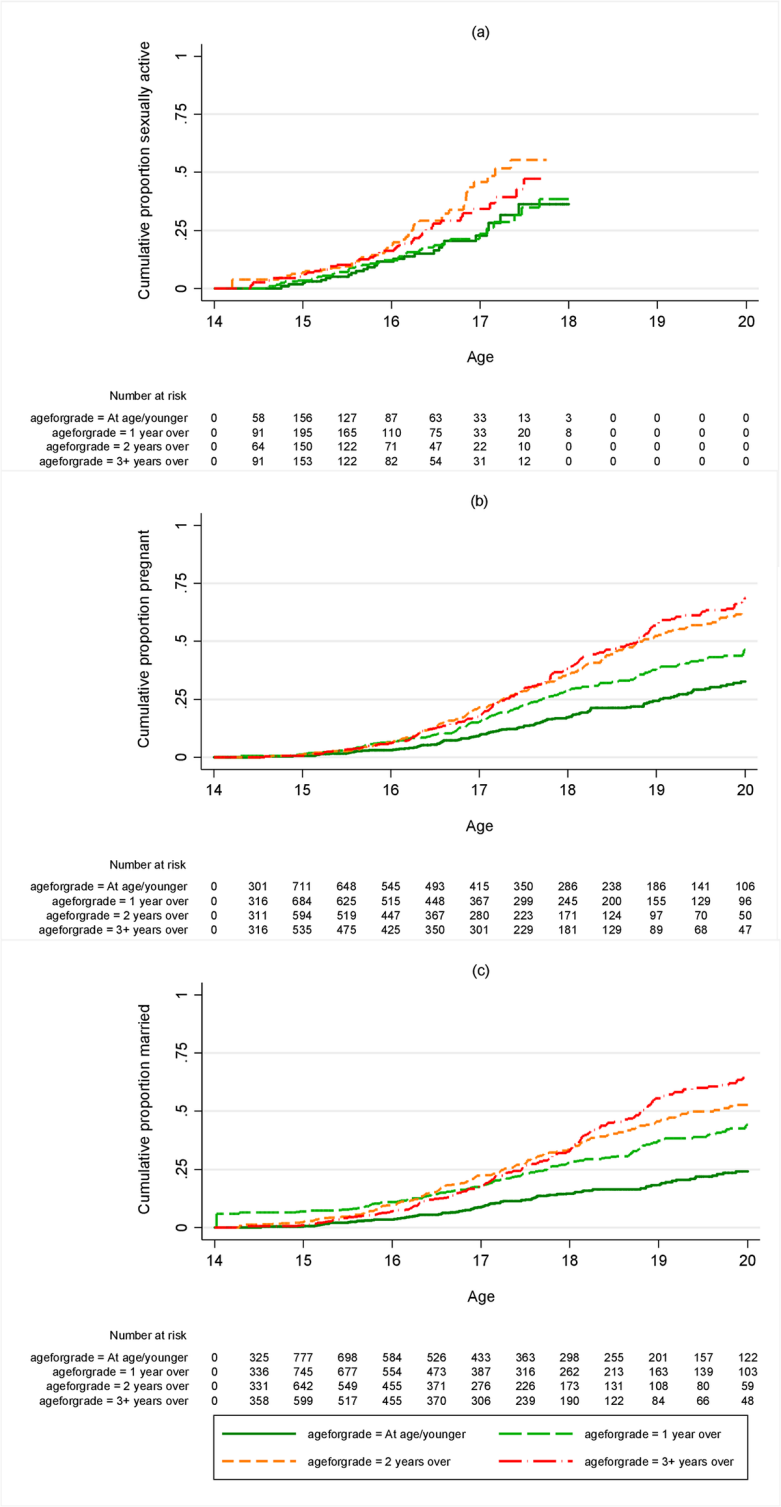
Cumulative proportion ever (a) sexually active (b) pregnant or (c) married by age for grade of girls at landmark age 14. Restricted to those who were in school at age 14 and not yet had the outcome in question.

**Fig 4:**
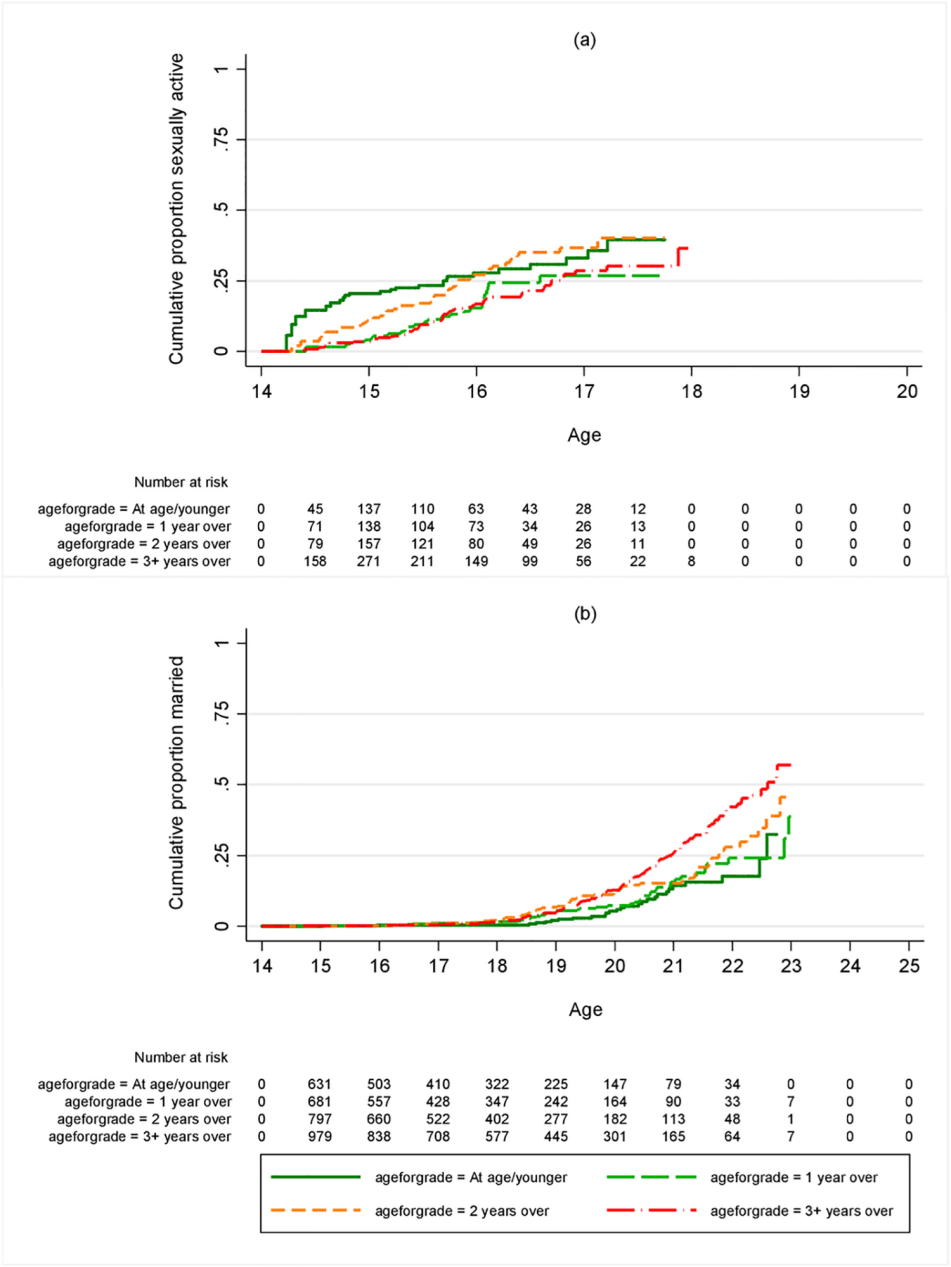
Cumulative proportion ever (a) sexually active (b) married, by age for grade of boys at landmark age 14. Restricted to those who were in school at age 14 and not yet had the outcome in question.

Figs 1–4 show the cumulative proportion of study participants with sexual debut, first pregnancy and first marriage by schooling status and age-for-grade at landmark age 14, separately for girls and boys. Similar figures for landmark ages 10–18 are in S1–S6 Figs. Tables 1–4 show the Cox regression analyses, with and without adjustment for confounders.

**Table 1:** Associations between schooling status and time to sexual debut, pregnancy and marriage for girls at different landmark ages. Hazard ratios (HR) and 95% confidence intervals (CI) are shown, compared to those in primary school.

Landmark age	Event/No. at risk	Crude HR (95%CI)			*P* (LRT)	Adjusted HR (95%CI)^1^			*P* (LRT)
		Dropped out in primary	Dropped out beyond primary	In secondary		Dropped out in primary	Dropped out beyond primary	In secondary	
**Sexual debut**									
13	101/577	6.33 (3.5–11.6)	NA	0.40 (0.06–2.87)	<0.0001	6.29 (3.0–13.1)	NA	0.50 (0.07–3.78)	0.0001
14	167/817	5.07 (3.27–7.85)	17.4 (2.39–127.1)	0.70 (0.31–1.59)	<0.0001	5.39 (3.27–8.86)	27.6 (3.45–221.0)	0.75 (0.32–1.74)	<0.0001
15	169/812	5.96 (4.07–8.73)	9.19(1.26–67.1) [1]	0.74 (0.44–1.23)	<0.0001	3.50 (2.22–5.52)	6.30 (0.74–53.6)	0.85 (0.50–1.45)	<0.0001
16	147/633	5.87 (4.02–8.56)	4.92 (0.68–35.7)	0.74 (0.48–1.15)	<0.0001	3.61 (2.21–5.87)	1.5 (0.19–11.9)	0.79 (0.49–1.26)	<0.0001
17	116/461	6.47 (4.07–10.28)	3.98 (1.66–9.52)	0.95 (0.58–1.54)	<0.0001	3.39 (1.90–6.07)	2.41 (0.92–6.32)	1.01 (0.60–1.68)	0.0002
18	66/295	9.88 (4.48–21.8)	8.41 (3.42–20.6)	0.66 (0.28–1.57)	<0.0001	8.32 (3.2–21.6)	6.62 (2.32–18.9)	0.63 (0.24–1.67)	<0.0001
**Pregnancy**									
13	645/2680	1.79 (1.21–2.65)	NA	0.28 (0.13–0.64)	<0.0001	1.52 (1.01–2.28)	NA	0.43 (0.19–0.96)	0.01
14	743/2508	2.85 (2.20–3.69)	37.1 (5.14–267.2)	0.35 (0.23–0.52)	<0.0001	2.39 (1.82–3.12)	55.9 (7.58–411.9)	0.42 (0.28–0.63)	<0.0001
15	770/2196	3.95 (3.27–4.76)	4.11 (1.95–8.66)	0.44 (0.34–0.57)	<0.0001	2.89 (2.35–3.46)	3.94 (1.82–8.52)	0.52 (0.40–0.67)	<0.0001
16	690/1827	4.08 (3.44–4.84)	5.24 (2.95–9.33)	0.46 (0.37–0.58)	<0.0001	2.84 (2.33–3.47)	4.80 (2.63–8.76)	0.52 (0.42–0.65)	<0.0001
17	500/1423	4.38 (3.54–5.43)	3.62 (2.46–5.32)	0.55 (0.43–0.70)	<0.0001	2.87 (2.23–3.69)	3.06 (2.04–4.61)	0.60 (0.46–0.77)	<0.0001
18	325/1064	5.59 (3.82–8.19)	5.53 (3.60–8.51)	0.75 (0.50–1.13)	<0.0001	3.87 (2.58–5.83)	4.28 (2.72–6.71)	0.75 (0.49–1.13)	<0.0001
**Marriage**									
13	604/2989	2.13 (1.46–3.12)	NA	0.21 (0.08–0.56)	<0.0001	1.79 (1.20–2.66)	NA	0.31 (0.12–0.84)	0.002
14	669/2744	3.06 (2.32–4.05)	NA	0.26 (0.16–0.41)	<0.0001	2.76 (2.08–3.67)	NA	0.31 (0.19–0.51)	<0.0001
15	658/2325	3.75 (3.04–4.63)	1.21 (0.39–3.75)	0.33 (0.25–0.44)	<0.0001	3.32 (2.67–4.12)	1.56 (0.49–4.89)	0.40 (0.30–0.55)	<0.0001
16	556/1913	3.67 (3.01–4.46)	1.70 (0.81–3.61)	0.39 (0.30–0.49)	<0.0001	2.99 (2.43–3.67)	2.30 (1.07–4.92)	0.44 (0.34–0.56)	<0.0001
17	373/1502	3.82 (2.99–4.87)	2.49 (1.62–3.82)	0.42 (0.32–0.56)	<0.0001	3.09 (2.38–4.02)	2.75 (1.74–4.33)	0.49 (0.37–0.64)	<0.0001
18	232/1133	6.55 (4.26–10.1)	4.03 (2.48–6.55)	0.61 (0.39–0.97)	<0.0001	4.67 (2.98–7.32)	3.58 (2.17–5.90)	0.72 (0.45–1.15)	<0.0001

Restricted to those with no missing data.

NA—Not available (insufficient data); LRT—Likelihood ratio test

^1^ Adjusted for education of parents, vital status of parents, living arrangements (household size, number of children aged 0–5 years in household, living with parents), sex of head of household, socioeconomic status (as five levels from principal component analysis of household assets), year of interview

**Table 2:** Associations between schooling status and time to sexual debut and marriage for boys at different landmark ages. Hazard ratios (HR) and 95% confidence intervals (CI) are shown, compared to those in primary school.

Landmark age	Event/No. at risk	Crude HR (95% CI)			*P* LRT	Adjusted HR (95% CI) ^1^			*P* LRT
		Dropped out in primary	Dropped out beyond primary	In secondary		Dropped out in primary	Dropped out beyond primary	In secondary	
**Sexual debut**									
13	125/636	1.36 (0.56–3.34)[5]	NA	NA	0.27	0.97 (0.38–2.49)	NA	NA	0.96
14	150/858	1.64 (0.73–3.74	NA	0.47 (0.15–1.48)	0.18	1.92 (0.81–4.55)	NA	0.43 (0.13–1.41)	0.12
15	139/835	2.38 (1.25–4.54)	1.66 (0.23–11.92)	0.78 (0.40–1.54)	0.092	2.26 (1.16–4.43)	1.44 (0.18–11.42)	0.82 (0.40–1.69)	0.15
16	120/673	1.53 (0.80–2.93)	NA	0.69 (0.40–1.19)	0.16	1.35 (0.67–2.70)	NA	0.73 (0.41–1.30)	0.42
17	95/525	2.50 (1.49–4.19)	1.07 (0.15–7.74)	1.10 (0.67–1.79)	0.015	2.48 (1.39–4.42)	1.77 (0.23–13.76)	1.21 (0.72–2.04)	0.03
18	59/403	4.4 (1.78–6.50)	1.89 (0.44–8.14)	1.19 (0.63–2.23)	0.0030	3.80 (1.90–7.62)	2.01 (0.41–9.86)	1.16 (0.60–2.27)	0.0026
**Marriage**									
13	186/3209	2.03 (1.00–4.12)	NA	NA	0.20	1.72 (0.82–3.61)	NA	NA	0.097
14	279/3029	3.46 (2.17–5.140	NA	0.21 (0.053–0.86)	<0.0001	3.74 (2.28–6.11)	NA	0.26 (0.064–1.04)	<0.0001
15	382/2857	2.88 (2.09–3.96)	NA	0.29 (0.16–0.52)	<0.0001	3.08 (2.22–4.28)	NA	0.35 (0.19–0.64)	<0.0001
16	493/2661	2.60 (2.04–3.32)	3.45 (0.48–24.62)	0.47 (0.39–0.64)	<0.0001	2.67 (2.08–3.43)	7.08 (0.95–52.68)	0.56 (0.41–0.77)	<0.0001
17	574/2492	2.30 (1.89–2.79)	0.78 (0.25–2.430	0.47 (0.37–0.60)	<0.0001	2.35 (1.92–2.87)	0.95 (0.30–3.01)	0.50 (0.39–0.64)	<0.0001
18	597/2227	2.35 (1.95–2.84)	0.76 (0.42–1.40)	0.62 (0.50–0.76)	<0.0001	2.40 (1.98–2.91)	0.86 (0.46–1.58)	0.68 (0.55–0.84)	<0.0001

Restricted to those with no missing data

NA—Not available (insufficient data); LRT—Likelihood ratio test

^1^ Adjusted for education of parents, vital status of parents, living arrangements (household size, number of children aged 0–5 years in household, living with parents), sex of head of household, socioeconomic status (as five levels from principal component analysis of household assets), year of interview

**Table 3:** Associations between age-for-grade and time to sexual debut, pregnancy and marriage for girls at different landmark ages. Hazard ratios (HR) and 95% confidence intervals (CI) are shown, compared to those at the correct age for grade or younger.

Landmark age	Event/No. at risk	Crude HR (95% CI)				Adjusted HR (95% CI) ^1^			
		1 year overage for grade	2 years overage for grade	3+ years overage for grade	P (trend)	1 year over	2 years over	3+ years over	P (trend)
**Sexual debut**									
12	41/309	0.82 (0.38–1.81)	1.19 (0.52–2.74)	1.26 (0.48–3.32)	0.79	0.95 (0.41–2.20)	1.39 (0.57–3.39)	1.00 (0.32–3.10)	0.84
13	89/557	1.05 (0.58–1.91)	1.40 (0.78–2.50)	1.82 (1.00–3.34)	0.19	0.97 (0.53–1.80)	1.14 (0.61–2.13)	1.42 (0.73–2.77)	0.66
14	142/777	1.01 (0.62–1.65)	1.66 (1.02–2.71)	1.46 (0.90–2.38)	0.077	1.07 (0.65–1.77)	1.68 (1.01–2.79)	1.50 (0.89–2.52)	0.12
15	133/757	1.29 (0.71–2.35)	1.28 (0.68–2.36)	1.55 (0.86–2.77)	0.49	1.32 (0.72–2.45)	1.08 (0.56–2.11)	1.28 (0.68–2.42)	0.72
16	100/575	0.73 (0.34–1.59)	1.50 (0.84–2.70)	1.31 (0.74–2.32)	0.16	0.75 (0.33–1.72)	1.44 (0.76–2.72)	1.26 (0.66–2.42)	0.31
17	66/385	2.15 (0.76–6.03)	2.41 (0.88–6.58)	1.86 (0.72–4.78)	0.31	1.99 (0.69–5.77)	1.87 (0.66–5.34)	1.46 (0.54–3.94)	0.51
18	23/235	NA	NA	NA		NA	NA	NA	
**Pregnancy**									
10	235/2608	1.72 (1.29–2.30)	1.55 (1.06–2.26)	3.00 (1.46–6.17)	0.0002	1.54 (1.14–2.08)	1.32 (0.87–2.01)	2.84 (1.32–6.17)	0.0077
11	387/2937	1.61 (1.26–2.06)	1.79 (1.37–2.34)	2.04 (1.34–3.10)	<0.0001	1.42 (1.10–1.84)	1.58 (1.19–2.10)	1.58 (1.02–2.47)	0.0069
12	518/2789	1.28 (1.02–1.61)	1.85 (1.47–2.32)	1.93 (1.45–2.56)	<0.0001	1.15 (0.91–1.45)	1.55 (1.22–1.97)	1.59 (1.18–2.15)	0.0008
13	619/2622	1.39 (1.11–1.74)	1.88 (1.51–2.35)	2.07 (1.64–2.62)	<0.0001	1.26 (1.01–1.58)	1.56 (1.24–1.95)	1.68 (1.31–2.15)	0.0003
14	679/2408	1.62 (1.28–2.05)	2.39 (1.90–3.02)	2.68 (2.13–3.36)	<0.0001	1.52 (1.19–1.92)	2.19 (1.73–2.78)	2.28 (1.79–2.89)	<0.0001
15	627/2016	1.66 (1.24–2.21)	2.42 (1.82–3.21)	3.01 (2.29–3.96)	<0.0001	1.56 (1.16–2.08)	2.09 (1.56–2.79)	2.48 (1.86–3.31)	<0.0001
16	463/1547	1.08 (0.73–1.59)	1.95 (1.45–2.61)	2.59 (1.95–3.44)	<0.0001	1.06 (0.72–1.57)	1.78 (1.32–2.40)	2.16 (1.60–2.91)	<0.0001
17	258/1107	1.39 (0.86–2.26)	1.72 (1.06–2.77)	2.63 (1.74–3.96)	<0.0001	1.31 (0.80–2.14)	1.55 (0.95–2.53)	2.24 (1.46–3.44)	0.0002
18	117/759	1.55 (0.68–3.55)	2.11 (1.05–4.25)	2.70 (1.38–5.29)	0.0082	1.56 (0.67–3.61)	2.02 (0.99–4.12)	2.50 (1.23–5.06)	0.04
**Marriage**									
10	211/2805	1.77 (1.31–2.39)	1.50 (1.00–2.25)	3.55 (1.72–7.32)	0.0002	1.52 (1.10–2.08)	1.15 (0.74–1.79)	3.19 (1.47–6.94)	0.008
11	345/3219	1.73 (1.33–2.23)	1.72 (1.29–2.29)	2.39 (1.56–3.65)	<0.0001	1.49 (1.14–1.96)	1.44 (1.06–1.96)	1.80 (1.15–2.84)	0.0095
12	473/3073	1.38 (1.08–1.75)	1.91 (1.49–2.43)	2.00 (1.48–2.70)	<0.0001	1.2 (0.94–1.55)	1.54 (1.20–1.99)	1.57 (1.14–2.15)	0.003
13	576/2921	1.49 (1.18–1.88)	2.09 (1.66–2.63)	2.19 (1.71–2.80)	<0.0001	1.32 (1.04–1.67)	1.70 (1.34–2.15)	1.68 (1.30–2.19)	0.0001
14	615/2644	1.70 (1.32–2.20)	2.63 (2.05–3.38)	3.07 (2.41–3.92)	<0.0001	1.57 (1.21–2.03)	2.38 (1.84–3.07)	2.62 (2.02–3.39)	<0.0001
15	550/2199	2.17 (1.56–3.03)	3.22 (2.31–4.47)	4.12 (2.99–5.68)	<0.0001	2.01 (1.43–2.81)	2.75 (1.96–3.85)	3.33 (2.38–4.65)	<0.0001
16	401/1681	1.31 (0.84–2.03)	2.77 (1.97–3.88)	3.48 (2.50–4.84)	<0.0001	1.22 (0.78–1.90)	2.44 (1.73–3.44)	2.81 (1.99–3.99)	<0.0001
17	213/1241	1.61 (0.90–2.90)	2.28 (1.30–4.00)	4.04 (2.47–6.62)	<0.0001	1.51 (0.84–2.74)	2.05 (1.16–3.63)	3.40 (2.04–5.69)	<0.0001
18	84/861	1.17 (0.41–3.33)	2.65 (0.16–6.03)	2.81 (1.26–6.27)	0.0061	1.13 (0.39–3.28)	2.50 (1.08–5.78)	2.58 (1.11–6.03)	0.024

Restricted to those with no missing data

NA—Not available (zero events in baseline category)

^1^ Adjusted for education of parents, vital status of parents, living arrangements (household size, number of children aged 0–5 years in household, living with parents), sex of head of household, socioeconomic status (as five levels from principal component analysis of household assets), year of interview

**Table 4:** Associations between age-for-grade and time to sexual debut and marriage for boys at different landmark ages. Hazard ratios (HR) and 95% confidence intervals (CI) are shown, compared to those at age or younger.

Landmark age	Event/No. at risk	Crude HR (95% CI)			P (trend)	Adjusted HR (95% CI)^1^			P (trend)
		1 year over	2 years over	3+ years over		1 year over	2 years over	3+ years over	
**Sexual debut**									
12	75/388	0.55 (0.29–1.05)	0.98 (0.56–1.71)	0.54 (0.26–1.09)	0.098	0.41 (0.21–0.82)	0.54 (0.28–1.02)	0.24 (0.11–0.55)	0.0026
13	120/618	1.15 (0.68–1.96)	1.14 (0.69–1.89)	0.94 (0.57–1.55)	0.82	1.25 (0.72–2.19)	1.06 (0.62–1.81)	1.01 (0.59–1.74)	0.85
14	144/836	1.00 (0.57–1.74)	1.51 (0.91–2.49)	0.94 (0.58–1.54)	0.13	1.06 (0.60–1.87)	1.49 (0.88–2.51)	0.94 (0.56–1.58)	0.18
15	128/801	1.49 (0.65–3.43)	1.85 (0.82–4.18)	1.46 (0.67–3.20)	0.44	1.52 (0.64–3.59)	1.77 (0.75–4.15)	1.32 (0.57–3.07)	0.43
16	110/637	1.41 (0.51–3.90)	1.50 (0.65–3.45)	1.69 (0.77–3.68)	0.54	1.53 ((0.54–4.33)	1.40 (0.59–3.31)	1.61 (0.71–3.64)	0.66
17	74/468	0.45 (0.15–1.34)	0.70 (0.28–1.73)	0.61 (0.30–1.25)	0.48	0.40 (0.13–1.25)	0.68 (0.26–1.80)	0.54 (0.24–1.20)	0.37
18	39/335	1.11 (0.20–6.09)	1.32 (0.28–6.25)	0.93 (0.22–3.92)	0.86	1.02 (0.17–6.16)	0.97 (0.19–4.97)	0.89 (0.19–4.18)	0.99
**Marriage**									
10	22/3041	0.54 (0.19–1.55)	1.10 (0.39–3.12)	NA	0.42	0.49 (0.16–1.52)	0.89 (0.29–2.74)	NA	0.23
11	44/3497	1.02 (0.50–2.06)	0.99 (0.47–2.09)	0.30 (0.040–2.25)	0.54	0.92 (0.44–1.90)	0.92 (0.42–2.05)	0.26 (0.033–2.03)	0.48
12	111/3357	0.60 (0.32–1.15)	1.92 (1.20–3.09)	1.44 (0.83–2.52)	0.0003	0.60 (0.32–1.16)	1.83 (1.12–2.98)	1.30 (0.72–2.35)	0.0030
13	178/3145	0.90 (0.52–1.57)	2.23 (1.43–3.49)	2.06 (1.32–3.20)	<0.0001	0.85 (0.49–1.49)	1.98 (1.25–3.13)	1.94 (1.22–3.09)	0.0001
14	260/2950	1.34 (0.83–2.19)	1.80 (1.14–2.83)	2.50 (1.65–3.79)	<0.0001	1.33 (0.81–2.17)	1.74 (1.10–2.76)	2.41 (1.56–3.70)	<0.0001
15	339/2730	2.89 (1.31–6.34)	5.35 (2.47–11.59)	5.77 (2.71–12.26)	<0.0001	2.57 (1.17–5.67)	4.64 (2.13–10.10)	4.64 (2.16–9.97)	<0.0001
16	414/2448	1.86 (1.01–3.45)	2.22 (1.31–3.77)	3.50 (2.11–5.80)	<0.0001	1.79 (0.97–3.33)	1.90 (1.11–3.25)	2.90 (1.72–4.86)	<0.0001
17	425/2166	1.89 (1.01–3.52)	2.04 (1.12–3.72)	3.49 (2.08–5.85)	<0.0001	1.93 (1.03–3.61)	1.91 (1.04–3.50)	3.12 (1.84–5.31)	<0.0001
18	390/1780	1.60 (0.85–3.00)	1.52 (0.85–2.71)	2.59 (1.54–4.35)	<0.0001	1.50 (0.80–2.84)	1.37 (0.76–2.47)	2.36 (1.39–4.02)	<0.0001

Restricted to those with no missing data

NA—Not available (insufficient data)

^1^ Adjusted for education of parents, vital status of parents, living arrangements (household size, number of children aged 0–5 years in household, living with parents), sex of head of household, socioeconomic status (as five levels from principal component analysis of household assets), year of interview

For girls, rates of first sex, pregnancy and marriage were all much higher for those out of school than those in school, and the associations with schooling status were only slightly less strong after adjusting for confounders (Table 1). The proportion sexually active increased rapidly among those out of school at each age (Fig 1, S1 Fig). The proportions pregnant and married also increased quickly in the out-of-school population, though not as dramatically as the proportion sexually active. (Fig 1, S2 and S3 Figs).

For boys there was an increased hazard of sexual debut among those out of school from age 14 (Fig 2, S1 Fig, Table 2), with or without adjusting for confounders, although with lower hazard ratios than for girls. Fewer boys than girls were out of school at the younger ages. Marriage for boys was much later than for girls, and hence occurred at a lower rate, but the relative hazard of marriage among those who dropped out of primary compared to those still in primary was similar to that for girls for most landmark ages (Table 2, Fig 2, S3 Fig). At each landmark age, rates of pregnancy and, for both boys and girls, marriage, were lower among those in secondary school than among those still in primary school (Tables 1 and 2).

**Fig 5:**
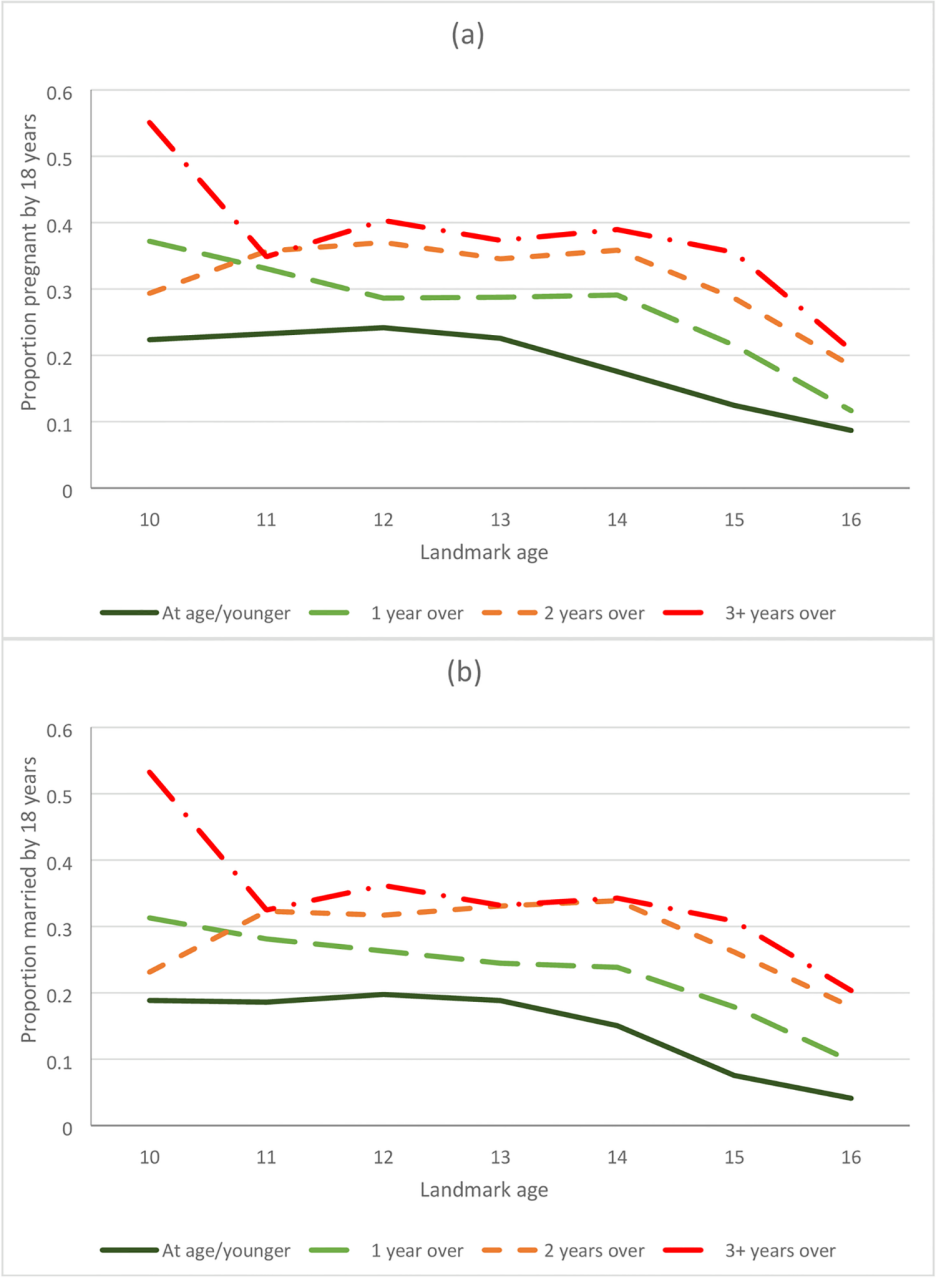
Proportion of girls (a) pregnant and (b) married before age 18, conditional on being in school and on school grade at different landmark ages.

There was no association between age-for-grade and sexual debut for girls or boys, except for boys at landmark age 12, among whom those not overage had a higher rate of sexual debut than those overage for their grade (Figs 3 and 4, S4 Fig, Tables 3 and 4). There were strong associations between age-for-grade and pregnancy and, for both boys and girls, between age-for-grade and marriage (Figs 3 and 4, S5 and S6 Figs, Tables 3 and 4). The associations with pregnancy and marriage were only slightly attenuated by adjusting for confounders. Additional adjustment for age at start of school made no difference to the results (not shown). The associations with pregnancy and marriage were similar at all ages and were apparent for girls from landmark age 10 onwards, although there were few pregnancies or marriages under 14. The proportion of girls pregnant before age 18 by age-for-grade is summarised in Fig 5a for different landmark ages. For example, of those ≥3 years behind at age 14, 39% were pregnant before they were 18, compared to 18% of those who were at or above the appropriate grade. The pattern for marriage was similar (Fig 5b). For boys there was insufficient follow-up time at the youngest ages to assess marriage rates accurately, since few boys marry under age 20, but an association between being overage for grade and earlier marriage was seen from the age of 12 onwards (Table 4).

## Discussion

In this large longitudinal population-based study, age-for-grade for those in school, as well as school drop-out, predicted age of pregnancy and marriage. Being out of school, but not age-for-grade, predicted sexual debut in girls, and, weakly, in boys.

A key insight from the landmark approach is that it allows us to see at what age being in or out of school or falling behind begins to impact on later life events. Up to age 13, almost all children were still in school so it was not possible to examine the effect of earlier dropout. For girls, associations of dropout with sex, pregnancy and marriage were already strong by age 13. For boys the association of dropout with marriage was strong by age 14. Many children were overage-for-grade, by age 10. By this age, girls who were three or more years behind were more likely to get pregnant or married early, even though these events were not imminent. For boys age-for-grade by age 12 was predictive of age at marriage: it was not possible to assess this at younger ages as the follow-up was not long enough.

The associations between being out of school and sexual activity, pregnancy and marriage are well recognised [7, 17, 26, 28]. The influence of age-for-grade on pregnancy and marriage may be because falling behind increases the risk of dropout. But, for girls, the rapidity with which sexual debut, pregnancy and marriage occur among those who are out of school at each age suggests that events leading to dropout may be important as well as actually being out of school. It is also possible that inaccurate dates due to reporting of events by year led to incorrect ordering of events in some cases. It is interesting that the associations with marriage were seen for boys as well as girls, albeit at older ages. Common factors underlie school progression, dropout and early sex, pregnancy and marriage [8]. We adjusted the analyses for available confounders and this had surprisingly little effect on the associations, but we were restricted by what was available. For example, academic aspirations of children and/or of their parents, which both influence and are influenced by performance [29], may be associated with dropout, pregnancy and marriage. We could only adjust for this indirectly through parental education level.

Children may be old for their grade because of late starts, temporary withdrawal, or grade repetition. In this population temporary withdrawal and late starts are rare. For example, among the girls in the analysis at landmark age 14, 92.2% had started at 6 years or younger, 6.6% started at 7 years and only 1.2% started at older than 7 years (S1 Table). Adjusting for starting age made no difference to the results. As most children were overage because of repetition, it is a reasonable proxy of performance, especially at primary school, which is free, so repetition is not caused by lack of money for school fees (although there may be other financial barriers). Decisions on repetition are made by teachers but, as elsewhere in sub-Saharan Africa, are often subjective and not based on standardised assessments [30]. In this population repetition is common in all grades [23].

The lack of association between age-for-grade and sexual debut at most ages may partly be due to the small sample size for this analysis, as information on sexual debut was only collected for a limited period and age group. Also, age at sexual debut may be more liable to problems of recall and reporting [25] than ages of pregnancy and marriage, which may have diluted any association. The higher risk of sexual debut at landmark age 12 for boys who were at or under age-for-grade may be due to chance, but could be explained by them mixing with older classmates [9, 13], as most children are already below the expected grade by this age [23].

Because landmark analysis defines exposures (and confounders) at a single point of time, it is different from looking at associations with the final education level or total years of schooling achieved [28, 31]. An alternative analytical approach would have involved a single Cox regression analysis where the exposure (school drop-out or age-for-grade) is treated as a time-varying variable. The confounders too would have to be time-varying, in particular vital status of parents, living arrangements, and household socioeconomic status. The interpretation of the estimated hazard ratios from such a model would rely on its implicit assumption of no feedback between time-varying exposure and time-varying confounders. As this is hard to justify, we have preferred the landmark approach as this breaks the analysis into overlapping time periods with time-fixed exposure and confounders, leading to more easily interpretable estimates of effects.

The landmark analyses performed at different ages are not independent, as individuals contribute to the analysis at each age at which they are seen and are still at risk of the outcome. The younger landmark ages, when few individuals will already have experienced the outcome, are more informative for the whole population than the older ages, which are applicable to the increasingly select group who have not yet experienced the outcome. However the similar hazard ratios at different landmark ages is striking. At each age, being in or out of school or the grade reached are important determinants of future life transitions.

Even though it was not possible totally to disentangle the effects of poor progression from its underlying causes, or to determine the extent to which poor progression influences the outcomes directly rather than through dropout and the loss of the “protective” effect of being in school, the results suggest that children at high risk of dropout and teenage pregnancy and marriage might be identified within the first few years of school. The solutions may correspondingly lie in the early childhood years. Teacher training and other pedagogic interventions can improve learning and school progression for some [32–34], though evidence for an effect on dropout or school completion is limited [32, 34]. They may also reduce teenage pregnancy and marriage.

## Supporting information

S1 Table Rates of outcomes by different exposures and potential confounders at landmark age 14 in girls. (DOCX)

S1 Fig Cumulative proportion ever sexually active, conditional on schooling status at landmark age. By landmark age and sex.
(DOCX)

S2 Fig Cumulative proportion ever pregnant, conditional on schooling status at landmark age. By landmark age.
(DOCX)

S3 Fig Cumulative proportion ever married, conditional on schooling status at landmark age. By landmark age and sex.
(DOCX)

S4 Fig Cumulative proportion ever sexually active, conditional on age-for-grade at landmark age. By landmark age and sex.
(DOCX)

S5 Fig Cumulative proportion ever pregnant, conditional on age-for-grade at landmark age. By landmark age.
(DOCX)

S6 Fig Cumulative proportion ever married, conditional on age-for-grade at landmark age. By landmark age and sex.
(DOCX)
