# Supplementary material for: Early school failure predicts teenage pregnancy and marriage: A large population-based cohort study in northern Malawi
Source: PLoS One. 2018 May 14;13(5):e0196041. doi: 10.1371/journal.pone.0196041 (PMC5951561; doi:10.1371/journal.pone.0196041)
Supplement: S1 Table — (DOCX) [file pone.0196041.s001.docx]

S1 **Table 1: Rates of outcomes by different exposures and potential confounders at landmark age 14 in girls**

|  |  | Sexual debut | | Pregnancy | | Marriage | |
| --- | --- | --- | --- | --- | --- | --- | --- |
|  |  | n | Rate/100PYAR  (95% CI) | n | Rate/100PYAR  (95% CI) | n | Rate/100PYAR  (95% CI) |
|  |  |  |  |  |  |  |  |
| Schooling | Out < primary | 26 | 60.8 (41.4-89.4) | 71 | 30.8 (24.4-38.9) | 60 | 28.6 (22.2-36.9) |
|  | Out ≥ primary | 1 | 144.5 (20.4-1000) | 1 | 144.5 (20.4-1000) | 0 |  |
|  | In primary | 139 | 13.2 (11.2-15.6) | 677 | 10.1 (9.4-10.9) | 616 | 8.7 (8.0-9.4) |
|  | In > primary | 6 | 8.6 (3.8-19.1) | 26 | 3.8 (2.6-5.6) | 17 | 2.3 (1.4-3.6) |
| Age for grade |  |  |  |  |  |  |  |
|  | At age | 28 | 10.6 (7.3-15.3) | 121 | 5.6 (4.7-6.6) | 100 | 4.3 (3.5-5.2) |
|  | I year over | 37 | 10.8 (7.8-14.9) | 177 | 8.8 (7.6-10.2) | 155 | 7.2 (6.-8.4) |
|  | 2 years over | 41 | 16.9 (12.4-22.9) | 194 | 12.0 (10.4-13.8) | 179 | 10.6 (9.2-12.3) |
|  | 3+ years over | 39 | 14.2 (10.4-19.4) | 211 | 15.6 (11.8-15.4) | 199 | 11.9 (10.4-13.7) |
| SES asset score |  |  |  |  |  |  |  |
|  | 1Poorest | 25 | 16.9 (11.4-25.0) | 123 | 14.4 (12.0-17.1) | 115 | 12.9 (10.7-15.4) |
|  | 2 | 33 | 17.0 (12.1-23.9) | 149 | 11.1 (9.5-13.1) | 121 | 8.4 (7.0-10.0) |
|  | 3 | 35 | 22.2 (16.0-31.0) | 134 | 11.7 (9.8-13.8) | 123 | 10.3 (8.7-12.3) |
|  | 4 | 37 | 14.0 (10.1-19.3) | 157 | 8.8 (7.5-10.3) | 145 | 7.6 (6.4-8.9) |
|  | 5 Richest | 41 | 10.2 (7.5-13.9) | 187 | 8.3 (7.2-9.6) | 169 | 7.1 (6.1-8.2) |
| Living with |  |  |  |  |  |  |  |
|  | Father only | 10 | 11.9 (6.4-22.1) | 52 | 12.0 (9.1-15.7) | 45 | 9.4 (7.0-12.6) |
|  | Mother only | 53 | 14.2 (10.8-18.5) | 216 | 10.2 (8.9-11.7) | 188 | 8.4 (7.3-9.7) |
|  | Both parents | 53 | 11.8 (9.0-15.5) | 285 | 8.6 (7.7-9.7) | 265 | 7.7 (6.8-8.7) |
|  | Neither parent | 56 | 21.4 (16.5-27.8) | 222 | 12.7 (11.1-14.5) | 195 | 10.3 (8.9-11.8) |
| Mother’s |  |  |  |  |  |  |  |
| education | None/<primary | 115 | 13.9 (11.6-16.7) | 570 | 10.6 (9.7-11.5) | 519 | 9.2 (8.4-10.0) |
|  | ≥ primary | 56 | 16.5 (12.7-21.4) | 203 | 9.2 (8.0-10.5) | 174 | 7.2 (6.2-8.3) |
| Father’s |  |  |  |  |  |  |  |
| education | None/<primary | 71 | 13.8 (11.0-17.5) | 378 | 11.5 (10.4-12.7) | 350 | 10.2 (9.2-11.4) |
|  | ≥ primary | 100 | 15.4 (12.7-18.7) | 394 | 9.1 (8.3-10.1) | 342 | 7.4 (6.6-8.2) |
| Mother alive |  |  |  |  |  |  |  |
|  | No | 23 | 25.3 (16.8-38.1) | 69 | 13.0 (10.3-16.5) | 58 | 10.2 (7.9-13.2) |
|  | Yes | 149 | 13.8 (11.8-16.2) | 706 | 10.0 (9.3-10.7) | 635 | 8.5 (7.8-9.2) |
| Father alive |  |  |  |  |  |  |  |
|  | No | 35 | 14.5 (10.4-20.1) | 138 | 9.7 (8.2-11.5) | 113 | 7.4 (6.1-8.9) |
|  | Yes | 135 | 14.6 (12.3-17.3) | 634 | 10.3 (9.5-11.1) | 577 | 8.8 (8.2-9.6) |
| Sex head |  |  |  |  |  |  |  |
| household | Female | 40 | 14.4 (10.6-19.7) | 177 | 9.7 (8.4-11.3) | 158 | 8.1 (7.0-9.5) |
|  | Male | 132 | 14.8 (12.5-17.6) | 598 | 10.3 (9.5-11.2) | 535 | 8.7 (8.0-9.5) |
| House hold |  |  |  |  |  |  |  |
| size | 1-5 | 60 | 17.6 (13.7-22.7) | 282 | 12.2 (10.8-13.7) | 246 | 10.1 (8.9-11.4) |
|  | 6-8 | 83 | 14.3 (11.5-17.7) | 365 | 9.2 (8.3-10.2) | 335 | 7.9 (7.1-8.8) |
|  | 9+ | 29 | 11.8 (8.2-16.9) | 128 | 9.7 (8.1-11.5) | 112 | 7.9 (6.6-9.5) |
| Children < 6 |  |  |  |  |  |  |  |
| In households | 0 | 67 | 16.0 (12.6-20.3) | 319 | 10.4 (9.3-11.6) | 288 | 9.0 (8.0-10.1) |
|  | 1 | 57 | 14.6 (11.2-18.9) | 260 | 9.7(8.6-11.0) | 220 | 7.6 (6.7-8.7) |
|  | ≥2 | 48 | 13.4 (10.1-17.8) | 196 | 10.6 (9.2-12.1) | 185 | 9.3 (8.0-10.7) |
| Age of mother |  |  |  |  |  |  |  |
| at birth | <20 | 36 | 18.8 (13.6-26.1) | 153 | 11.8 (10.1-13.9) | 130 | 9.4 (7.9-11.1) |
|  | 20-34 | 99 | 13.7 (11.2-16.7) | 464 | 9.5 (8.6-10.4) | 429 | 8.3 (7.6-9.1) |
|  | 35+ | 17 | 10.5 (6.6-17.0) | 89 | 10.6 (8.6-13.1) | 73 | 8.2 (6.5-10.4) |
| Age of father |  |  |  |  |  |  |  |
| at birth | <25 | 25 | 16.4 (11.1-24.3) | 135 | 11.9 (10.1-14.1) | 129 | 11.0 (9.2-13.0) |
|  | 25-34 | 63 | 15.1 (11.8-19.3) | 262 | 9.5 (8.4-10.7) | 245 | 8.5 (7.5-9.6) |
|  | 35+ | 44 | 12.0 (8.9-16.1) | 228 | 9.9 (8.7-11.2) | 200 | 8.1 (7.1-9.3) |
| Firstborn |  |  |  |  |  |  |  |
|  | No | 94 | 11.9 (9.7-14.6) | 498 | 9.7 (8.9-10.6) | 453 | 8.4 (7.7-9.2) |
|  | Yes | 58 | 20.3 (15.7-26.3) | 208 | 10.9 (9.5-12.4) | 179 | 8.8 (7.6-10.1) |
| Dwelling score |  |  |  |  |  |  |  |
|  | 1Poorest | 25 | 20.1 (13.6-29.7) | 79 | 17.1 (23.7-21.3) | 67 | 13.5 (10.6-17.1) |
|  | 2 | 41 | 13.6 (10.0-18.5) | 133 | 14.0 (11.8-16.5) | 128 | 12.7 (10.7-15.1) |
|  | 3 | 30 | 18.3 (12.8-26.1) | 81 | 12.9 (10.3-16.0) | 73 | 10.5 (8.3-13.2) |
|  | 4 | 23 | 14.2 (9.5-21.4) | 67 | 12.3 (9.7-15.6) | 58 | 9.5 (7.4-12.3) |
|  | 5 Richest | 25 | 12.3 (8.3-18.1) | 82 | 9.3 (7.5-11.5) | 64 | 6.4 (5.0-8.2) |

| (continued) |  | Sexual debut | | Pregnancy | | Marriage | |
| --- | --- | --- | --- | --- | --- | --- | --- |
|  |  | n | Rate/100PYAR  (95% CI) | n | Rate/100PYAR  (95% CI) | n | Rate/100PYAR  (95% CI) |
|  |  |  |  |  |  |  |  |
| Age at school |  |  |  |  |  |  |  |
| start | <6 | 33 | 13.0 (9.2-18.2) | 170 | 8.4 (7.2-9.7) | 148 | 6.9 (5.9-8.1) |
|  | 6 | 130 | 15.6 (13.1-18.5) | 543 | 10.9 (10.0-11.8) | 488 | 9.2 (8.4-10.1) |
|  | 7 | 9 | 12.0 (6.2-23.1) | 55 | 11.4 (8.8-14.9) | 49 | 9.6 (7.2-12.6) |
|  | 8+ | 0 |  | 7 | 11.9 (5.7-25.0) | 8 | 13.0 (6.5-26.0) |

n= number of events; SES= socioeconomic status
