# Supplementary material for: Early school failure predicts teenage pregnancy and marriage: A large population-based cohort study in northern Malawi
Source: PLoS One. 2018 May 14;13(5):e0196041. doi: 10.1371/journal.pone.0196041 (PMC5951561; doi:10.1371/journal.pone.0196041)
Supplement: S3 Fig — By landmark age and sex. (DOCX) [file pone.0196041.s004.docx]

**S3 Fig. Cumulative proportion ever married, conditional on schooling status at landmark age. By landmark age and sex.**

The numbers at risk are shown under each graph. Note different scales on the x-axes.

Number at risk

Dropped out of primary

In primary

In secondary

Number at risk

Dropped out of primary

In primary

In secondary

Number at risk

Dropped out of primary

In primary

In secondary

Number at risk

Dropped out of primary

Dropped out of secondary

In primary

In secondary

Number at risk

Dropped out of primary

Dropped out of secondary

In primary

In secondary

Number at risk

Dropped out of primary

Dropped out of secondary

In primary

In secondary

Dropped out of primary

In primary

Dropped out of secondary

In secondary

Dropped out of primary

In primary

Dropped out of secondary

In secondary
