# Supplementary material for: Early school failure predicts teenage pregnancy and marriage: A large population-based cohort study in northern Malawi
Source: PLoS One. 2018 May 14;13(5):e0196041. doi: 10.1371/journal.pone.0196041 (PMC5951561; doi:10.1371/journal.pone.0196041)
Supplement: S4 Fig — By landmark age and sex. (DOCX) [file pone.0196041.s005.docx]

**S4 Fig. Cumulative proportion ever sexually active, conditional on age-for-grade at landmark age. By landmark age and sex.**

The numbers at risk are shown under each graph. Note different scales on the x-axes.
